# Supplementary figures and images for: Evidence for vagal sensory neural involvement in influenza pathogenesis and disease
Source: PLoS Pathog. 2024 Apr 16;20(4):e1011635. doi: 10.1371/journal.ppat.1011635 (PMC11051609; doi:10.1371/journal.ppat.1011635)

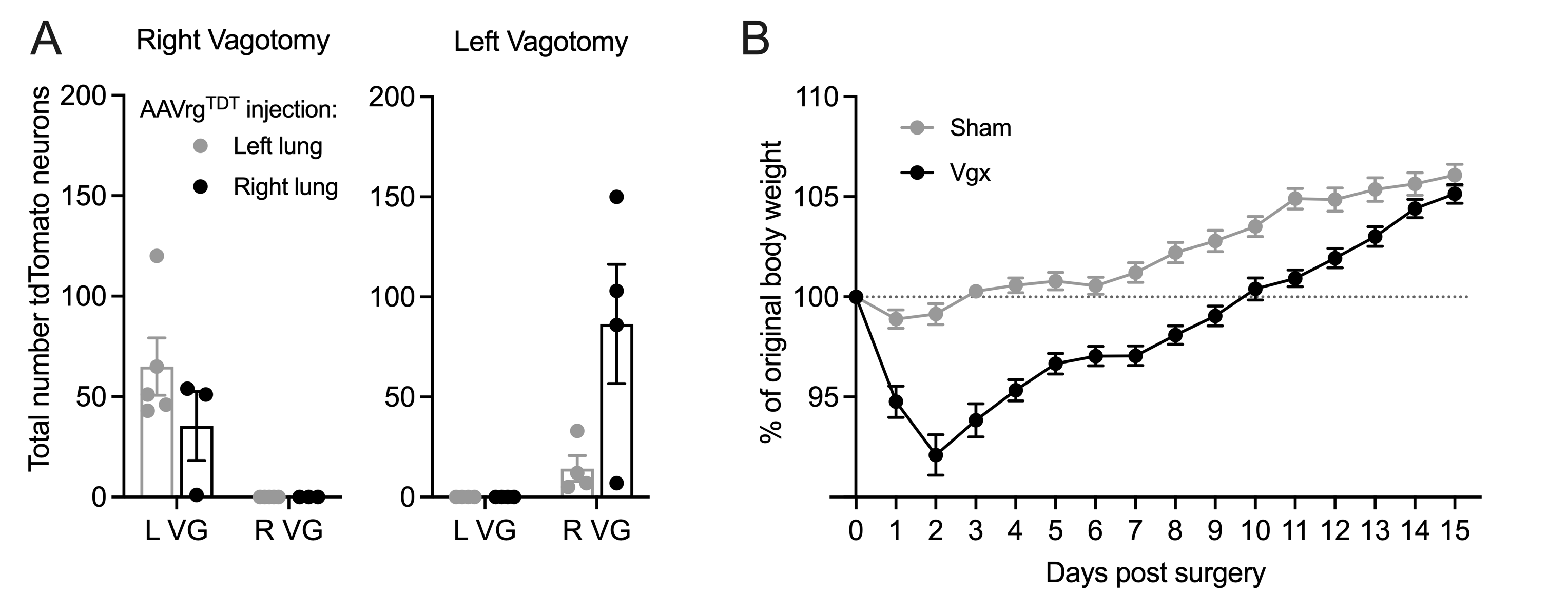

Supplement: S1 Fig — Graphs depict (A) quantification of the total number of tdTomato+ neurons (retrogradely labelled from either left or right lung lobes) in either left or right vagal sensory ganglia (VG) following unilateral left or right vagotomy and (B) body weight change post vagotomy or sham surgery and prior to IAV or mock infection. Data represented as mean ± SEM. (TIFF) [file ppat.1011635.s001.tiff]

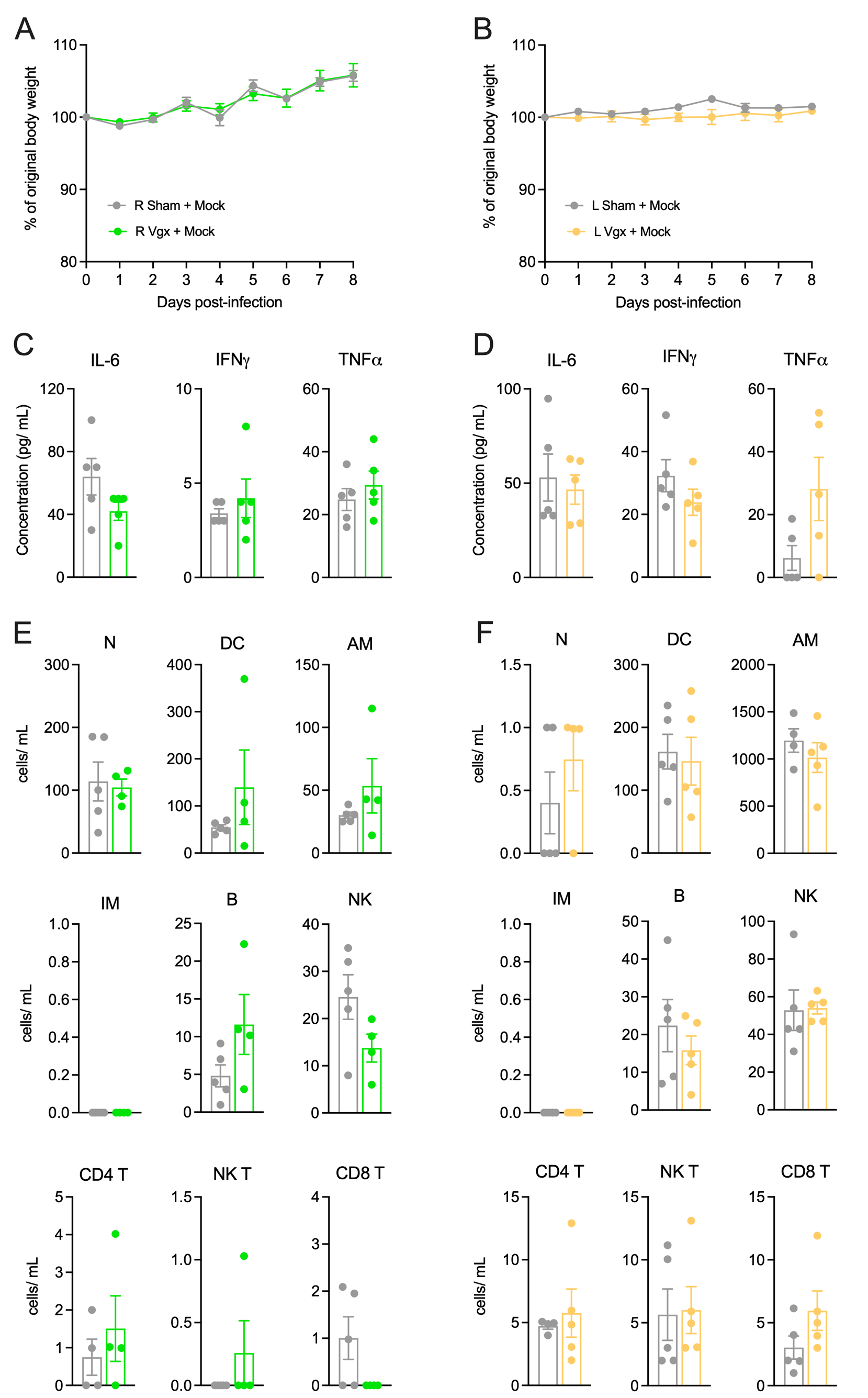

Supplement: S2 Fig — Graphs depict body weight change, lung cytokine measurements and lung immune cell populations in (A, C, E) right and (B, D, F) left vagotomized/ sham mice following intranasal mock (PBS) inoculation. N = 5 for each group at 8 days post infection. Data represented as mean ± SEM. (TIFF) [file ppat.1011635.s002.tiff]

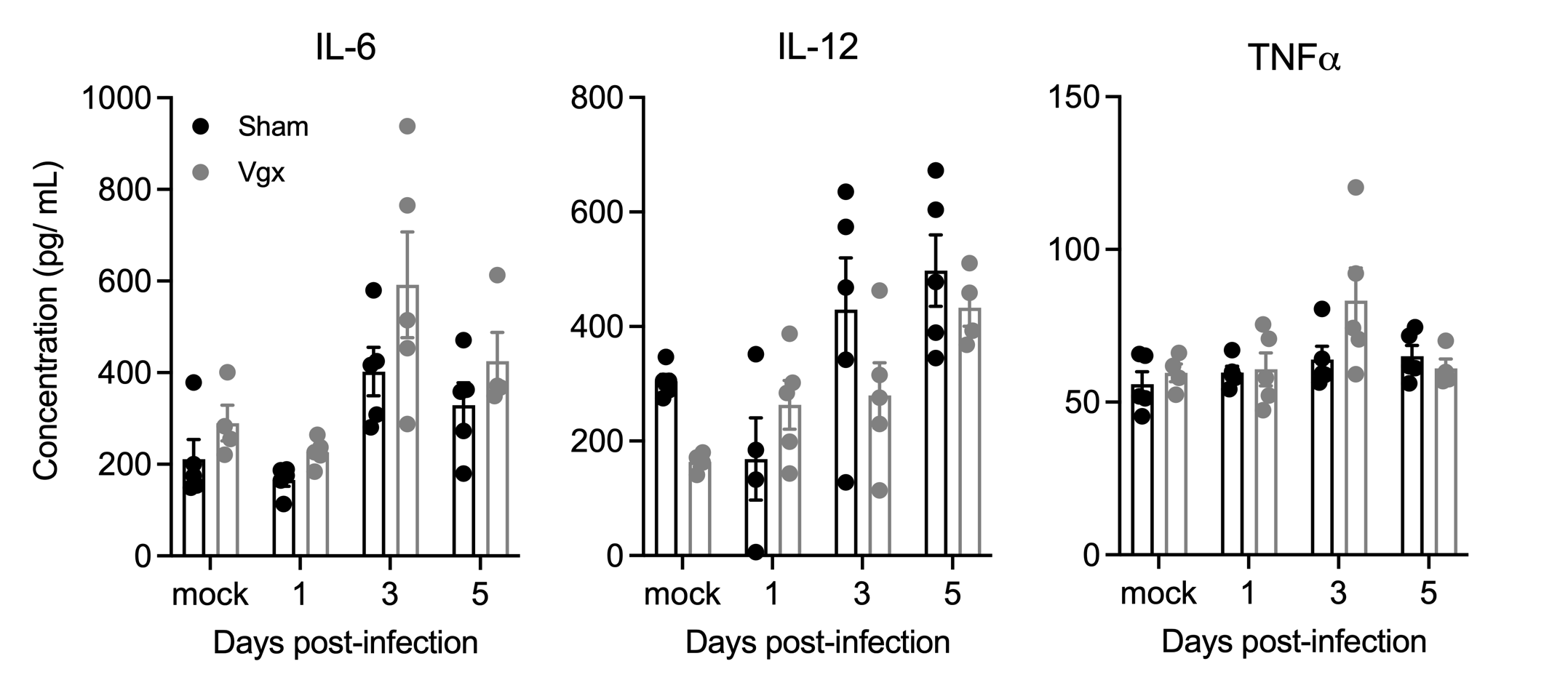

Supplement: S3 Fig — Graphs depict cytokine measurements taken from vagotomized and sham mice following IAV or mock infection (day 5). N = 5 for each group. Data represented as mean ± SEM. (TIFF) [file ppat.1011635.s003.tiff]

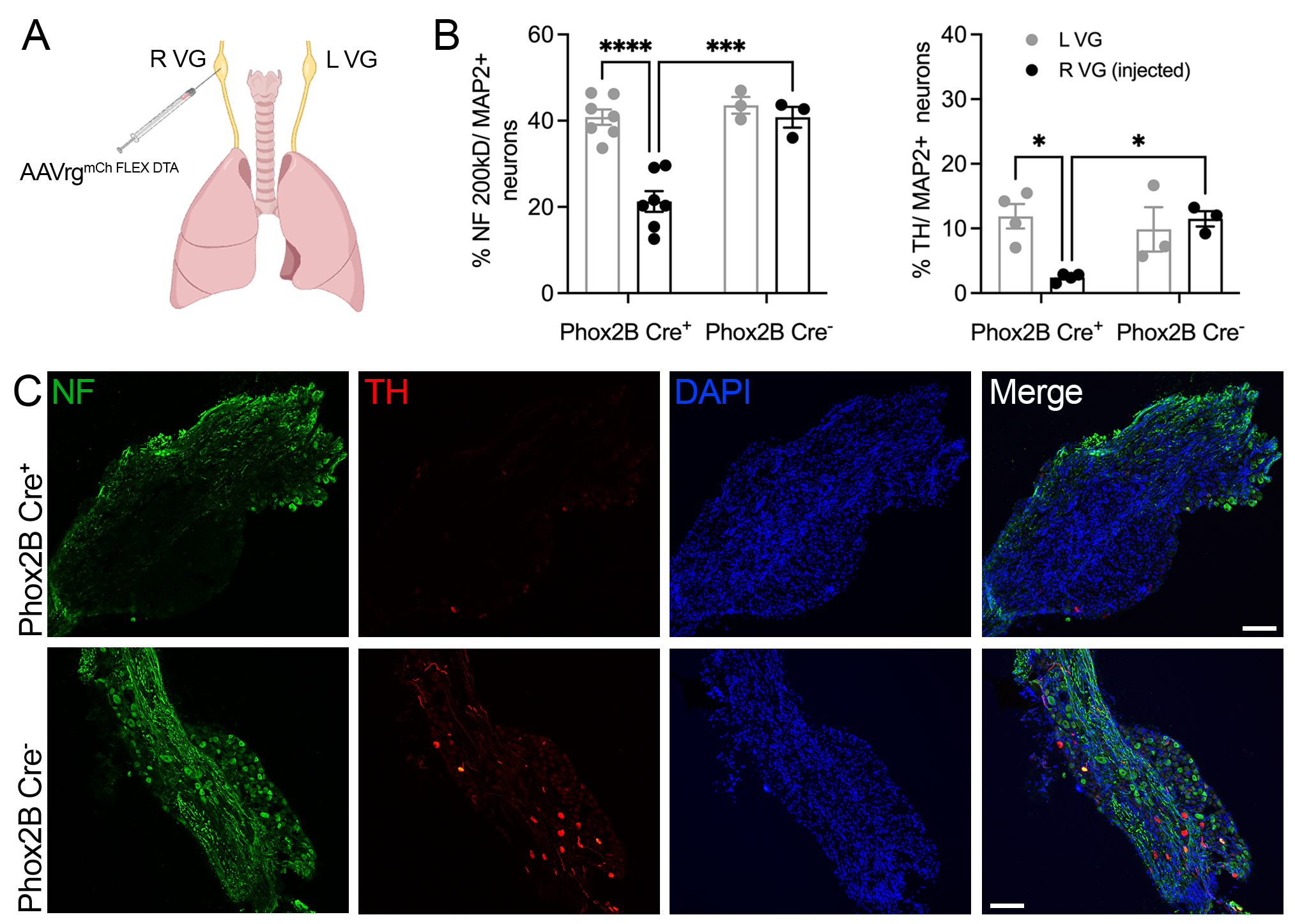

Supplement: S4 Fig — (A) Schematic showing intraganglionic injection of genetic ablation of sensory neurons in the vagal sensory ganglia AAVrgmCh FLEX DTA in Phox2B-Cre+ (n = 7) and Phox2B-Cre- (n = 3) mice. (B) Graphs depict the percentage of (A) neurofilament (NF) 200kD and (B) tyrosine hydroxylase (TH) -expressing neurons per the total number of neurons (MAP2+) in either right (R; injected side) or left (L; non injected side) vagal ganglia (VG) and images (C) demonstrate immunostained vagal ganglia sections from the right vagal ganglia from either Phox2B-Cre+ or Phox2B-Cre- mice following 4 weeks of microinjection of AAVrgmCh FLEX DTA. Data represented as mean ± SEM. *, ***, **** denotes significance of p < 0.05, p < 0.001, p < 0.0001, respectively, as (B) determined by mixed-effects analysis corrected for multiple comparisons (Šídák). Scale bar represents 100μm. Cartoons were created with BioRender.com. (TIF) [file ppat.1011635.s004.tif]

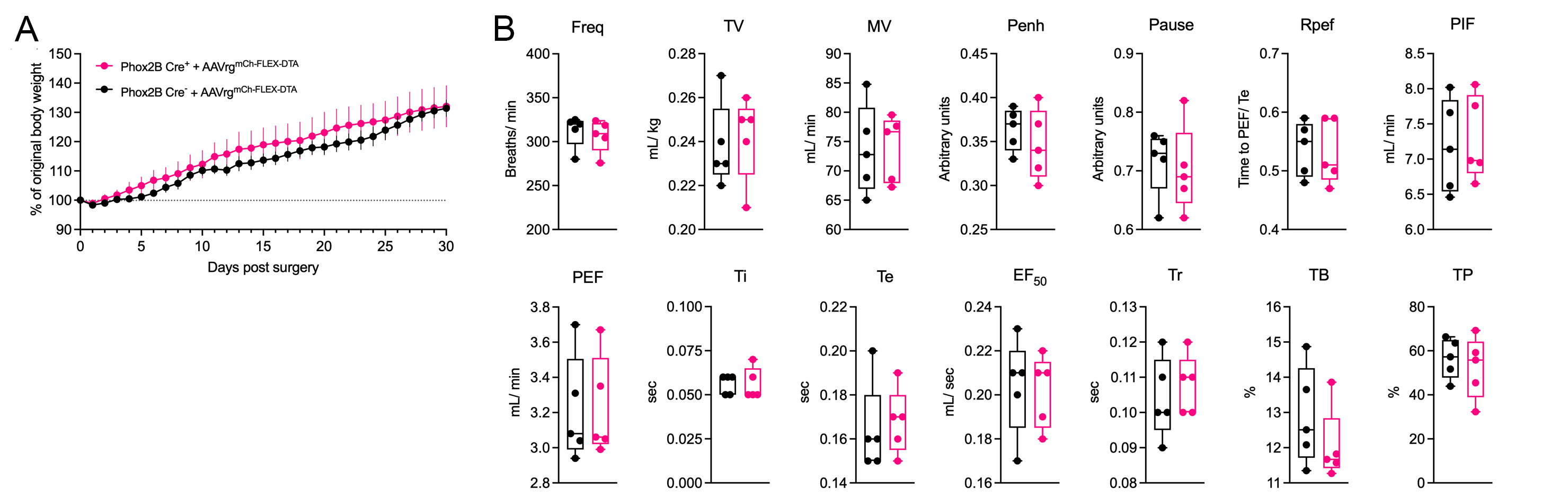

Supplement: S6 Fig — Graphs depict (A) body weight change post-surgery after injection of AAVrgmCh FLEX DTA into the lungs of Phox2B-Cre+ or Phox2B-Cre- mice and (B) respiratory parameters measured 30 days post-surgery (prior to IAV infection; n = 5 per group). Data represented as mean ± SEM. Freq, frequency; TV, tidal volume; MV, minute volume; PENH, enhanced pause; PAU, pause; Rpef, location into expiration where the peak occurs (PEF) as a fraction of Te; PIF, peak inspiratory flow; PEF, peak expiratory flow; Ti, inspiratory time; Te, expiratory time; EF50, expiratory flow at 50% expired volume;Tr, relaxation time; TB, duration of breaking; TP, duration of pause before expiration. (TIF) [file ppat.1011635.s006.tif]

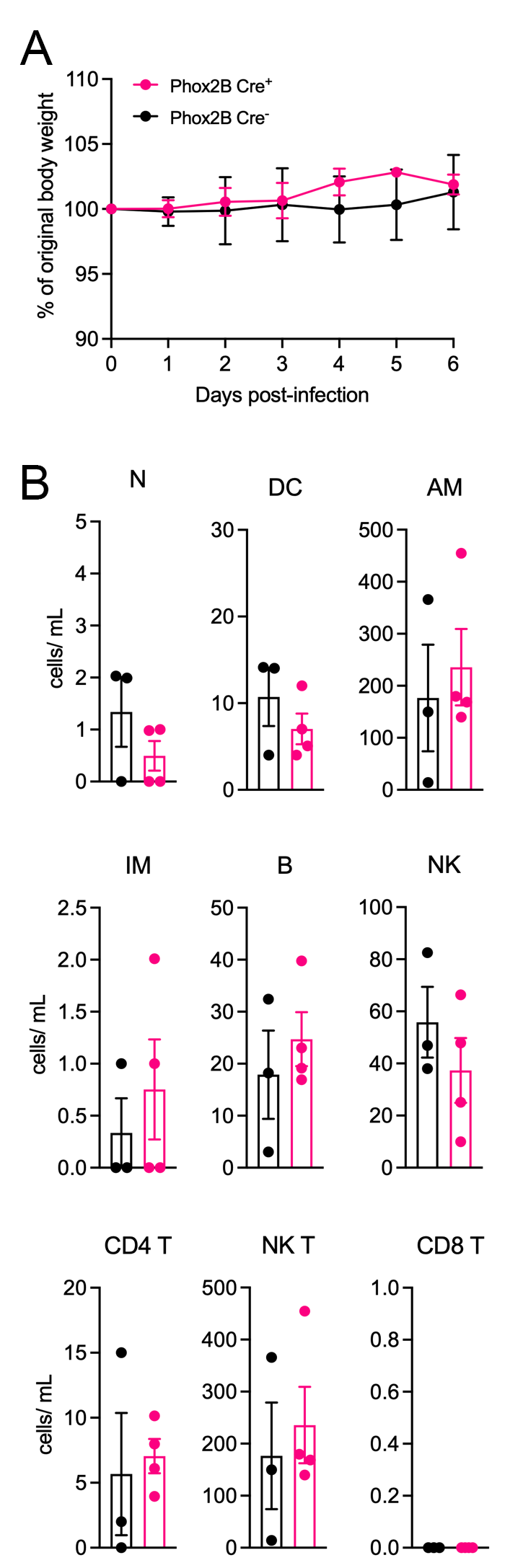

Supplement: S7 Fig — Graphs depict (A) body weight change and (B) lung immune cell populations (Day 6 post-infection) in Phox2B-Cre+ and Phox2B-Cre- mice following intranasal (PBS) inoculation. N = 3–5 per group. Data represented as mean ± SEM. (TIF) [file ppat.1011635.s007.tif]

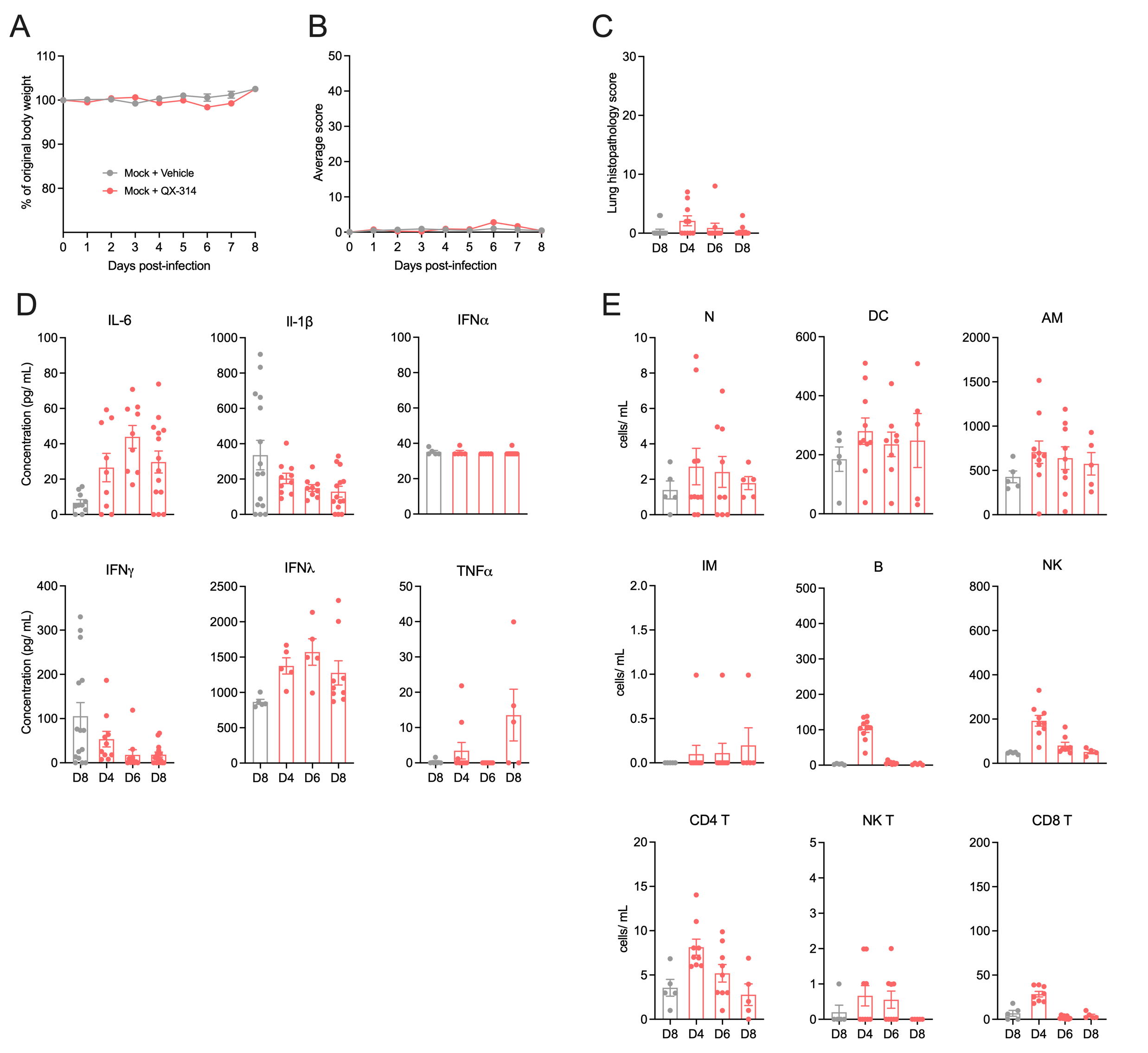

Supplement: S8 Fig — Graphs depict (A) body weight change, (B) clinic scoring, (C) lung histopathology, (D) lung cytokine and € lung immune cell population measurements in mice receiving nebulized 300 μM QX-314 or vehicle (saline) following intranasal mock (PBS) inoculation. N = 5–10 for each group at days 4, 6, 8 post infection. Data represented as mean ± SEM. N, neutrophils; DC, dendritic cells; AM, alveolar macrophages; IM, interstitial macrophages; B, B cells; NK, natural killer cells. (TIFF) [file ppat.1011635.s008.tiff]

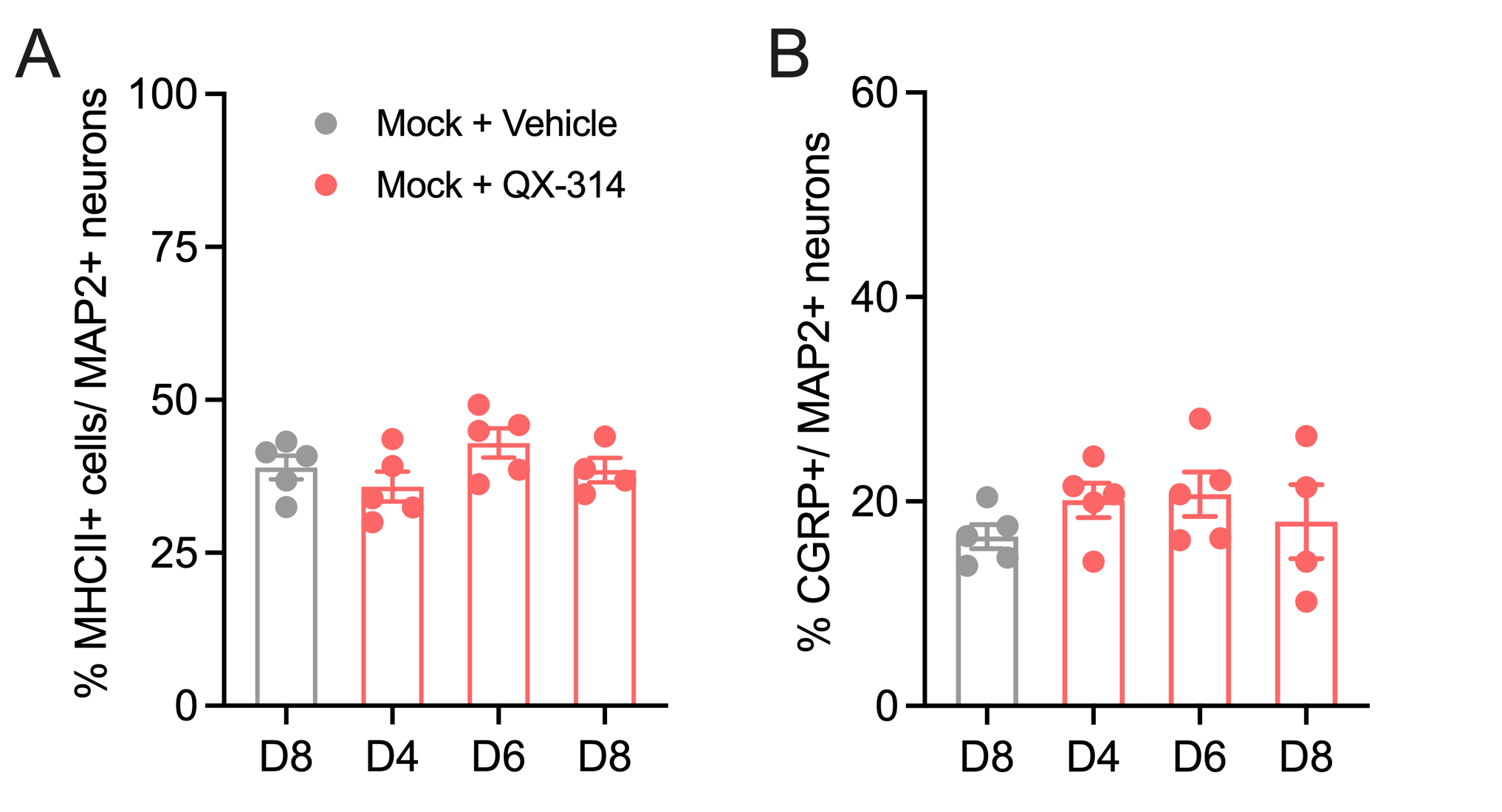

Supplement: S9 Fig — Graphs depict the percentage of (A) MHC II-expressing cells and (B) CGRP-expressing neurons per the total number of neurons in the vagal sensory ganglia in mice receiving nebulized 300 μM QX-314 or vehicle (saline) following intranasal mock (PBS) inoculation. N = 5 for each group at days 4, 6, 8 post infection. Data represented as mean ± SEM. (TIFF) [file ppat.1011635.s009.tiff]

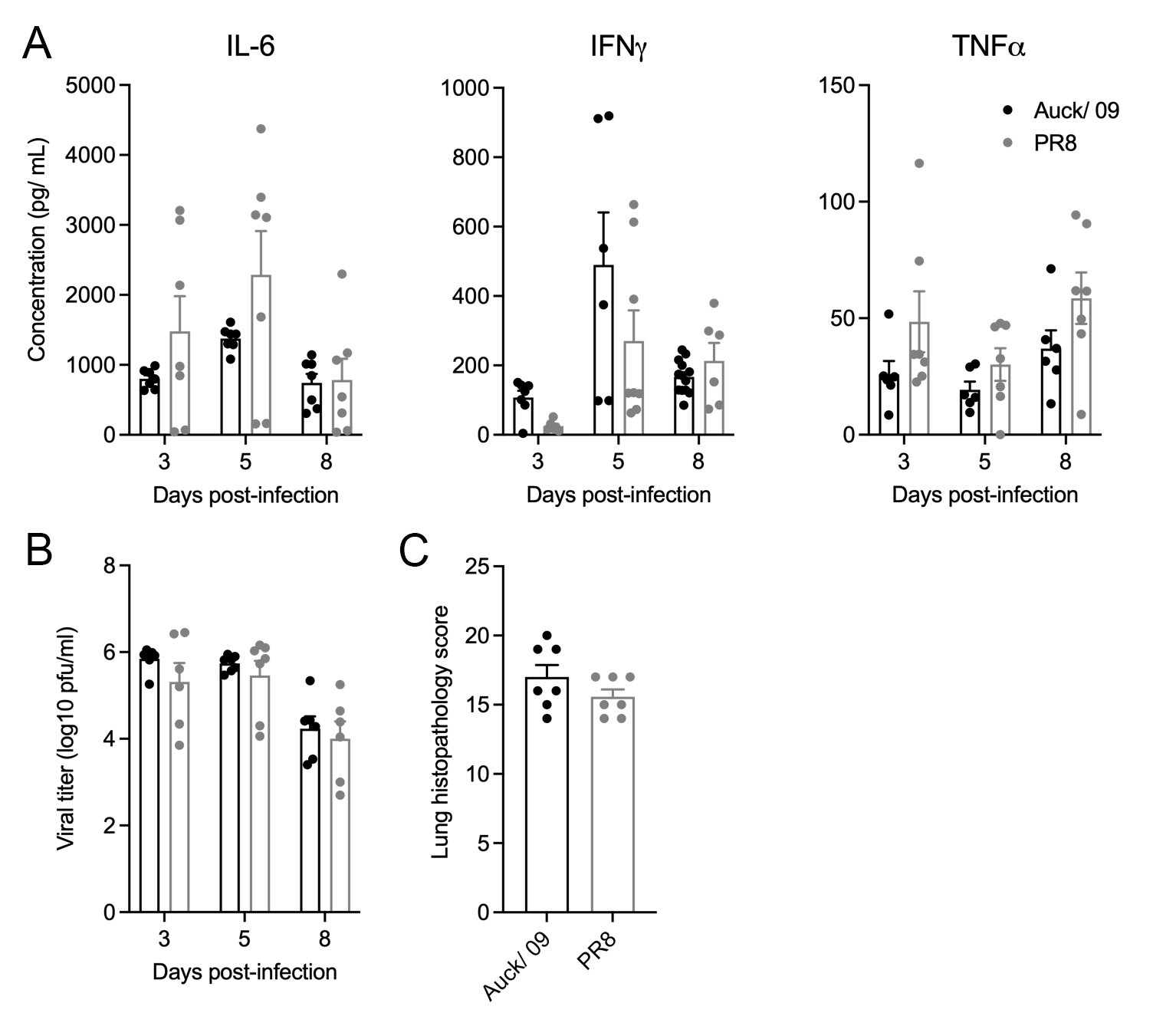

Supplement: S10 Fig — Graphs depict (A) lung cytokines, (B) lung viral titers, and (C) lung histopathological score post infection with either Auck/ 09 or PR8 H1N1 viral strains. N = 5–7 for each group at 3, 6, 8 days post infection. Data represented as mean ± SEM. (TIF) [file ppat.1011635.s010.tif]

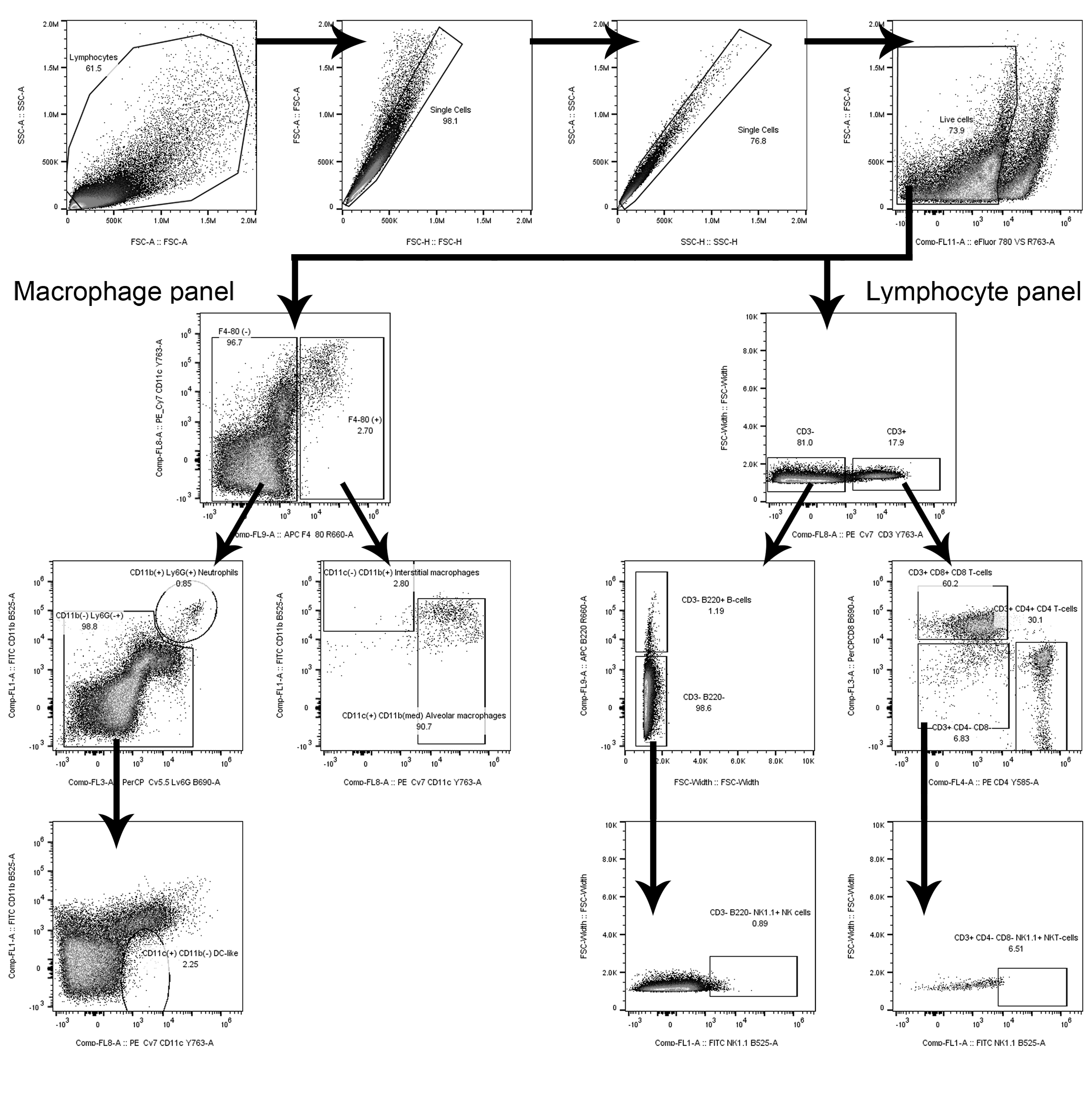

Supplement: S11 Fig — Representative flow cytometry plots used to identify immune cell populations in mouse bronchoalveolar lavage fluid (BALF). (TIF) [file ppat.1011635.s011.tif]
