# Supplementary material for: Evidence for vagal sensory neural involvement in influenza pathogenesis and disease
Source: PLoS Pathog. 2024 Apr 16;20(4):e1011635. doi: 10.1371/journal.ppat.1011635 (PMC11051609; doi:10.1371/journal.ppat.1011635)
Supplement: Attachment — Submitted filename: Response to reviewers.docx [file ppat.1011635.s013.docx]

We would like to thank the reviewers for their insightful reviews, critique and feedback on our manuscript. Considering these comments, we have made significant changes to manuscript, including new experiments and data, the rearrangement of figures and notable additions to the discussion. We hope these new additions and changes have greatly increased the manuscripts readability and interest. Please see below our comments to the reviewers in blue. Thank you**.**

**Part I - Summary**
*Please use this section to discuss strengths/weaknesses of study, novelty/significance, general execution and scholarship.*

**Reviewer #1:** The manuscript by Verzele et al. on the role of vagal sensory innervation of the lung during influenza infection provides some unique insight into the role of neuroimmune circuits in the lung. In general, the manuscript is well written, and is obviously a substantial amount of work that is presented. General comments for the authors to improve the manuscript include a revision to integrate some of these data better. For example, the wonderful tracing studies presented in Figure 1 are not prominently mentioned in the discussion with any interpretation or indication as to what these mean for the current study and going forward.

Thank you for the complimentary comments. The purpose of the tracing experiments was to determine the relative contribution of left and right vagus nerves to the sensory innervation of the left and right lungs (and therefore determine which vagus nerve to surgically disrupt). We have made edits to the manuscript to better integrate this data.

The data on the immune cells and cytokines is similarly presented in my view. I didn’t get a feeling for what the changes in how the authors believed the different populations contributed to any changes. Personally, I did not like the many tables in this version and would have preferred separate graphs for each cell population so that one can easily see changes over time. Other suggestions include the removal of the precise numbers in the actual text it simply repeats what is in the figure and for me disrupts the flow and decreases readability. In the end, I’m not sure what is the proposed mechanism here.

Thank you for the suggestions to help with the readability of the manuscript. We have removed the tables and numbers from the text and have presented the lung immune cell populations and other data in graph format. We have attempted to clarify our interpretation of the lung inflammatory marker changes in the revised manuscript.

Specific questions are listed as minor and major with either the associated figure number or text line number.

**Reviewer #2:** The manuscript under review uses a mouse model of Influenza A virus to investigate how ablation of the vagal nerve or ablation of lung sensory neurons or inhibition of pulmonary sensory neurons impacts on the course of influenza A disease and inflammatory and immune responses.
They find a complex picture where vagotomy and ablation of pulmonary vagal sensory neurons has almost no effect on pulmonary inflammation yet worsens clinical disease and inhibition of pulmonary sensory neurons decreases lung pathology and worsens clinical disease.

Overall, this is a well-designed study using a very interesting approach. The methods are described thoroughly and the paper is well-written. While this is certainly an interesting study, I have concerns about the success rate of the methods to manipulate vagal innervation and about the interpretation of the results.

1) Overall the effects are rather marginal and one wonders if the used methods to manipulate vagal innervation are really working efficiently. For the complete vagal denervation, the authors say that this led to a transient reduction in body weight (data not shown). Why are these data not shown? In the world of paperless publication, there is no reason for not showing these data. This also applies to the data for the spread of the AAV. In humans, vagotomy (which in the past was done also in the non-gastro selective way) leads to continuous weight loss. Why should this be different in mice? Could it be that there is reinnervation, which would put the murine vagotomy paradigm under stress?

In our experience, when a unilateral vagotomy is performed in healthy (non-obese) animals they generally recover quite well. We have now added a supplementary figure to show the time course of this recovery (body weight – Supp Figure 1B). The data in humans demonstrates that a unilateral vagotomy results in prolonged body weight loss generally due to a suppression of calorie intake, however these experiments are performed in humans that are morbidly obese, not in humans of a healthy weight. Perhaps this is the source of the different outcomes between murine and human observations. In terms of reinnervation, as a rule, when we perform cervical vagotomies in mice we remove at least a 1cm portion of the nerve to prevent reinnervation occurring. To confirm this, we have added data (Supp Figure 1A) showing that unilateral vagotomy (performed 2-3 weeks earlier) eliminated retrograde neuron tracer expression in neurons in the ipsilateral ganglia following lung injection. The spread of lung AAV was visually assessed by an experienced investigator and documented as ‘evidence of spread’ or ‘no evidence of spread’ (outside of the lung). As such, there is no formal data to present for this measure and we retain the ‘data not shown’ statement on this occasion. In our past experience from several retrograde lung tracing experiments, if tracer enters unintentionally enters into the pleural cavity this shows up as hundreds of traced neurons in the vagal ganglia – beyond the number of neurons that innervate the lungs.

2) In Fig. 1, D and G, the vagatomized mice with Influenza A lose more weight than the sham-operated ones. Could this be due to the vagal denervation per se. To exclude this one would need a group of mice with vagatomy alone.

We performed a vagotomy or sham surgery and then mock infection to show this and this data was included in the first version of the manuscript as Supp Fig 1. We have retained this data in the revised submission as Supp Figure 2.

Vagotomy mice following mock infection didn’t not lose weight in comparison to sham + mock animals. The same is true for the mice shown in Fig. 1L where ablation of pulmonary vagal sensory neurons is achieved by genetic tools.

We have now performed additional experiments to show that a mock infection in Phox2B-Cre^+^ and Phox2B-Cre^-^ plus AAV DTA doesn’t result in weight loss in animals where pulmonary vagal sensory neuron ablation has occurred (Supp Fig 7).

3) In the discussion, the authors correctly state that:” These observations suggest that pulmonary sensory neurons may play complex and multifaceted roles in respiratory disease, perhaps specific to the underlying pathogen or disease pathology.”I think all of us would agree with that. The question what does this paper add to unravel this complex and multifaceted role

The extensive revisions of the manuscript hopefully clarify this point for the reviewer. The neuroinflammatory events in the vagal ganglia are a significant and novel finding and we assert that these may be an important and underappreciated component of IAV pathogenesis and associated disease morbidity. In addition to this, for the sake of transparency we would like to point out that we found a cut and paste error in the vagal ganglia qPCR data which occurred when transferring the raw data from an excel file into the prism file. This has now been corrected and has slightly changed some of the values however has not changed the story.

**Part II – Major Issues: Key Experiments Required for Acceptance**
Please use this section to detail the key new experiments or modifications of existing experiments that should be absolutely required to validate study conclusions.

Generally, there should be no more than 3 such required experiments or major modifications for a "Major Revision" recommendation. If more than 3 experiments are necessary to validate the study conclusions, then you are encouraged to recommend "Reject".

Reviewer #1: Table 1, How are there interstitial macrophages in the BALF?, The gating strategy presented in figs5 does not seem to take note that both alveolar and interstitial macrophages express CD11c, with alveolar macrophages being CD64+ (or f480+) CD11b-/low CD11c+ SiglecF+, with interstitials typically CD64+ CD11b+ CD11c+ SiglecF-. I also noticed that the plots provided and gating strategy appear to be showing the height (H) not area (A) for each fluorphore. This certainly is not standard and makes further interpretation difficult. I did not find how these gates were set, and if a FMO control was used on the BALF. In my view the population after the F4/80 gate is likely all alveolar macrophage, and that SiglecF should be used here to discern these. I agree the CD11b staining is quite high and am not sure if this is biologically real, a consequence of the height display, or simply a detector voltage issue. Please see PMC3824047 table 1, and associated figures for other widely used gating strategies. Is there a reason why only BALF was assessed and not whole lung?

Table 1. I’m not sure what population is the most important in the author's mind or the phenomenon being described. For the significant populations, it would help to include these in a figure and have the rest of the data in a table. I would like to be able to cross-compare more quickly and visually these different data sets across time.

Thank you for the noticing this mistake and for the suggestions. We have reanalysed the flow data to resolve the query. The gating was changed from height to area, overall changing the plots including these for alveolar and interstitial macrophages. The gating was then adjusted, based on the FMO controls and the negative control (see figure below). The adjustments in the gating strategy changed the cell count of the infiltrating inflammatory cells and have been corrected throughout the paper. Additionally, the tables have been converted to graphs to allow for an easier comparison between the populations.

The flow cytometry is performed on the BALF only, and not on whole lung. Both approaches allow to study the influx of inflammatory cells within the pulmonary system. We appreciate that each approach has advantages and disadvantages and will inform different aspects of lung pathology. As our workflow was designed to collect the lungs for a variety of endpoints measurements, we opted to work with the BALF for cell counts.

In regard to SiglecF, the reviewer is of course correct as it is used readily in mice to differentiate alveolar from interstitial macrophages. From the outset we began these experiments assuming that we wouldn’t see huge populations of interstitial macrophages as we were using a mild dose of flu which doesn’t cause severe lung damage and we were collecting the BALF (interstitials are difficult to collect from the BALF unless significant lung damage has occurred). Therefore, we didn’t feel it were necessary to rely on SiglecF and instead opted to use Cd11b and Cd11c to define those populations. We do however feel that in future experiments we will opt to use SiglecF as a marker and perhaps analyse whole lung instead of BALF.

Figure 1 Gating strategy based on A) FMO minus CD11c, B) FMO minus CD11b, C) applied to a full stained sample.

Fig1 Please include H&E figures even in supplementary data so that readers and reviewers can see these data.

We have now added in two new figures including examples of H&E scoring parameters (Supp Figure 12) and a breakdown of the scores and representative images of QX-314 experiments at days 4, 6, 8 post infection (Fig 7).

Fig1/Text starting at line 153: The strategy to ablate Phox2B+ lung innervation is an interesting approach. Can you describe more about how you validate that less mCherry is really due to loss of these cells? Can you perform ISH or confocal for phox2B+ and count cells in the NTS? The concern is that the lack of a virally encoded signal may not reflect that it is working as you expect. Was there any change in mouse body weight after virally mediate ablation of neurons in the NTS or is this really restricted and without effect on any other neurons and functions within the NTS?

The AAV construct we used expresses mCherry in transduced neurons that do not express Cre and, in the presence of Cre, only DTA (not mCherry) is expressed. Hence, a reduction in the number of mCherry positive neurons in the vagal sensory ganglia and reduced nerve terminal density in the NTS (where the pulmonary vagal neurons project to centrally) of Phox2B-Cre^+^ mice compared to Phox2B-Cre^-^ mice is consistent with successful DTA expression. Counting the number of mCherry neurons in the vagal sensory ganglia, like the reviewer suggests, doesn’t provide insight into the cellular ablation abilities of the DTA, it only informs us that the construct is likely behaving the way it should. To address these concerns, we performed several additional experiments:

Firstly, we attempted Phox2B immunohistochemistry and couldn’t get the antibody to work convincingly in the vagal sensory ganglia, perhaps because Phox2B is more prominently expressed during neural development.

Instead, we opted to inject the AAV construct directly into the vagal ganglia, which will target any Phox2B expressing neuron, not just pulmonary afferents. Subsequently, we then performed immunohistochemistry for neurofilament (NF) 200kD and tyrosine hydroxylase (TH), proteins expressed by subsets of nodose ganglia neurons. Cell counts of these immunomarkers revealed a significant reduction in the number of NF 200kD and TH neurons in the vagal sensory ganglia on the injected side of Phox2B-Cre^+^ mice compared to Phox2B-Cre^-^ mice (Supp Fig 4). In these same animals we found a reduction of purinergic P2X2 receptor labelling in the NTS in Phox2B-Cre^+^ mice, an indicator that the vagal nerves have been lesioned and their nerve terminals in the brainstem have degenerated (Supp Fig 5). Interestingly, we also observed a significant reduction in the number of choline acetyl transferase (CHAT) positive neurons in the dorsal motor nucleus of the vagus on the injected side in Phox2B-Cre^+^ mice. This was likely due to uptake of the AAV construct via the axons of the vagal preganglionic motor neurons that traverse through the vagal sensory ganglia (Supp Fig 5).

Collectively, these data suggest that the AAV construct is functioning correctly, and cellular ablation is occurring as hoped.

Finally, we’ve added a new figure that demonstrates that body weight doesn’t change in mice that have undergone cellular ablation of pulmonary vagal afferents. In addition, respiratory parameters remain unchanged i.e. cellular ablation does not seem to impact baseline respiratory functions (Supp Fig 6).

Fig 1. With the virally mediated neuronal depletion, are there any changes in the vagus that would occur due to infection with your viral vector? Can you describe what was done to mitigate this possibility or is the virally transduced phox2B.Cre- mouse completely normal?

We don’t see any impact of AAV transduction in Phox2B-Cre mice and the IAV induced pathology in these animals is indistinguishable from wildtype mice not transduced with AAVs. But we understand the limitations of this approach, which in part explains the rationale of employing multiple interventions to interrogate the study questions.

Please indicate the time post-infection in the figure for the phox2b.Cre LSL-DTA mice experiments, also I didn’t see this information in table 4. Thank you for picking up on this. These details have now been added.

Again, there are indications in the results at line 178 about reduced pathology without the actual H&E as well. These have been added in as requested above.

Line 184/fig 2 for the QX314 studies, is there evidence that TRPV1 is only expressed on neurons especially during viral infection? Is there any data on this already in the literature?

There is evidence that TRPV1 can be expressed by non-neuronal cells, including but not limited immune cells (Saunders et al., Mol Immunol, 2007) and respiratory epithelial cells (Reilly et al., J Biochem and Mol Tox, 2005). However, such cells are not known to express appreciable (if any) NaV1.8, the target of QX-314. The absence of any profound effect on pulmonary pathology of QX-314 in IAV infected animals and the absence of any notable effects in mock infected animals appears to be consistent with minimal off-target effects of the drug. However, for completeness, a new limitations section has been added to mention off target possibilities.

Line 195. Nebulization of the drug was clearly intended to allow for exposure in the airway, is there some possibility that nebulized drug was ingested during animal grooming and would this alter your conclusions? Can you estimate the dose (ug/Kg of body weight) that the animals received?

There is the possibility that the drug was ingested during nebulization due to animals grooming. QX-314 only exerts its effects intracellularly. Therefore, it needs access to the intracellular environment of the neurons and the way it does this is through TRP channels (in particular TRPV1 and TRPA1). Besides capsaicin, some products of inflammation act as TRP channel agonists allowing the channels to open and QX-314 to access the internal environment of the neuron. The inflammation caused by IAV respiratory infection is likely contained to the lung environment, therefore by nature it is unlikely QX-314 is impacting gut neurons. Nevertheless, again, this possibility has been mentioned in the limitations section.

In the initial submission of the manuscript, we reported the estimated dose of QX-314 as 0.04 mg/kg. This was determined via the algorithm proposed by Alexander et al., Inhal Toxicol, 2008. The possible oral dosage is very difficult to estimate, but conceivably will be much lower than this.

Tables 5. These are some of the most interesting data that could use additional explanation in the discussion and how these fit with the ideas of neuronal regulation of pathology.

These data are now featured in new Figure 5. Some additional discussion has been added to explore these data further.


In Fig S2 why is vehicle group for all days of infection not shown?

We ran a small preliminary experiment to determine there was no difference between vehicle groups over that short course of time. Therefore, to comply with the reduction component of ethical guidelines we opted to run one vehicle group that was culled at day 8 and compared the experimental groups to that.

In Fig 3. Please put some group/figure keys on the graphs.

Apologies for missing that, they have been added.

In the text at line 227 in describing cytokines is this in the lung or in the BALF. Will these two compartments match?

The cytokine measurements are made from the lung only and not the BALF.

Is there a role for IFNa/b or lambda here?

This is an interesting point and we have now performed some extra experiments looking at both IFNa and lambda (Fig 5). We didn’t find any difference between the vehicle and QX-314 groups. So, it appears there may not be a significant role for these interferons.

Again tables of flow cytometry, the questions about gating strategy need to be addressed.

We have addressed this as per above comment.

Line 249: This portion of the manuscript seems to be the focus of the authors, at this point it seems almost like the immune cell data and other measures are not as well integrated with the description of the effects on the nodose presented beginning at line 249. I also appreciate that Isg15 is an interferon stimulated gene, however, is this really because of the measured IFNg or that IFNa/b/l, that was not measured, is driving this expression?

This is an interesting question and as mentioned above, no differences were observed between vehicle and QX-314 groups for IFNa and lambda. Therefore, it remains possible that IFNy is driving Isg15 expression.

Discussion. I think the sentence at line 287 that summarizes the work, misses some nuance. Perhaps I missed it but, I do not see the histopathology so I don’t know if I agree that pathology was slightly increased in denervation. Some aspects of clinical presentation were not changed such as body weight. These statements in my view are therefore an overstatement or that the data doesn’t seem to be presented.

Thank you for this comment. We have taken the opportunity to revise the statement and include a more nuanced overview of the study findings.

Reviewer #2: see above

**Part III – Minor Issues: Editorial and Data Presentation Modifications**
Please use this section for editorial suggestions as well as relatively minor modifications of existing data that would enhance clarity.

Reviewer #1: Minor issues:
Line 127 Is the lung viral titer from the whole lung, Would there be differences in left vs. right lung lobe titers in mice with right or left vagotomy. Is there a hyper-local response to a viral pathogen or challenge?

This is an insightful question. Initially, we thought the same and ran a series of experiments not part of this study assessing this and found no difference between left vs right lung. Therefore, we opted to measure lung viral titers from the whole lung.

Line 144: There is no difference in the three cytokines measured would be a statement that better reflects the data, not that systemic cytokines are the same.

Thank you. This has been changed.

Line 146, 149 on line 146 there is an indication that the table values are x10^3, Is this correct, and confirm that this is correct for all the other tables?

We have now placed all these datasets onto graphs which may make the data more readable.

Line 518 flow gating is fig s5 not 4 as indicated. In this section you should better indicate how the gates were set. Are these FMO on BALF etc.

This has been addressed as per comments above.

Reviewer #2: see above
